# Nighttime sleep duration, 24-hour sleep duration and risk of all-cause mortality among adults: a meta-analysis of prospective cohort studies

**DOI:** 10.1038/srep21480

**Published:** 2016-02-22

**Authors:** Xiaoli Shen, Yili Wu, Dongfeng Zhang

**Affiliations:** 1 Department of Epidemiology and Health Statistics, Medical College of Qingdao University, Shandong, Qingdao 266021, Dongzhou Road, No.38, P.R. China

## Abstract

A dose-response meta-analysis was conducted to summarize evidence from prospective cohort studies about the association of nighttime sleep duration and 24-hour sleep duration with risk of all-cause mortality among adults. Pertinent studies were identified by a search of Embase and PubMed databases to March 2015. A two-stage random-effects dose–response meta-analysis was used to combine study-specific relative risks and 95% confidence intervals [RRs (95% CIs)]. Thirty-five articles were included. Compared with 7 hours/day, the RRs (95% CIs) of all-cause mortality were 1.07 (1.03–1.13), 1.04 (1.01–1.07), 1.01 (1.00–1.02), 1.07 (1.06–1.09), 1.21 (1.18–1.24), 1.37 (1.32–1.42) and 1.55 (1.47–1.63) for 4, 5, 6, 8, 9, 10 and 11 hours/day of nighttime sleep, respectively (146,830 death cases among 1,526,609 participants), and the risks were 1.09 (1.04–1.14), 1.05 (1.02–1.09), 1.02 (1.00–1.03), 1.08 (1.05–1.10), 1.27 (1.20–1.36), 1.53 (1.38–1.70) and 1.84 (1.59–2.13) for 4, 5, 6, 8, 9, 10 and 11 hours/day of 24-hour sleep, respectively (101,641 death cases among 903,727 participants). The above relationships were also found in subjects without cardiovascular diseases and cancer at baseline, and other covariates did not influence the relationships substantially. The results suggested that 7 hours/day of sleep duration should be recommended to prevent premature death among adults.

Over the past 30 years, there has been an increasing interest in the association between sleep and health outcomes^1^. Sleep problems are common and are anticipated to increase with the rapid advent of the ‘24/7’ society involving round-the-clock activities and increasing night-time use of television and internet^1^,^2^. Previous studies showed that short duration of sleep is associated with increased risks of stroke, coronary heart disease, metabolic syndrome, hypertension, central adiposity, obesity and type 2 diabetes^2^,^3^,^4^,^5^,^6^,^7^,^8^, and long duration of sleep is also associated with increased risks of stroke, coronary heart disease, type 2 diabetes, and colorectal cancer^2^,^3^,^8^,^9^. These data suggest that sleep duration may be associated with the risk of all-cause mortality. While a previous systematic review^10^ indicated that both short and long duration of sleep are predictors of all-cause mortality among adults, the risk of all-cause mortality associated with specific duration of sleep has not been summarized, and the definition of short and long duration of sleep differed between studies which might complicate the interpretation of pooled results. In addition, results from recent prospective cohort studies^11^,^12^,^13^,^14^,^15^,^16^,^17^,^18^,^19^,^20^,^21^,^22^,^23^,^24^,^25^,^26^,^27^,^28^,^29^ on sleep duration and the risk of all-cause mortality among adults have not also been quantitatively summarized with a meta-analysis. Therefore, we conducted a dose-response meta-analysis following the PRISMA guidelines (Table S1) to quantitatively assess the effect of nighttime sleep duration and 24-hour sleep duration on the risk of all-cause mortality among adults, respectively.

## Results

### Study characteristics

The flow chart for study inclusion is shown in Figure S1. A total of 35 articles were included in this meta-analysis. Among the 35 articles, 14 were conducted in Asia, 11 in Europe, 8 in USA, 1 in Brazil and 1 in Australia. The duration of follow-up ranged from 2.8 to 25 years. Sleep duration was obtained by self-administered or interviewer-administered questionnaires in all studies but 1 study^29^ (sleep diaries were used). The mean age of participants ranged from 40.7 to 83.4 years. All studies adjusted for age and sex (or gender-specific results were provided). The reference category of sleep duration is 6–9 hours/day, but most of studies treated 7–8 hours/day as the reference category. The included studies met the quality score of 4 to 8 stars. For nighttime sleep duration, results from 25 articles with 36 results (gender-specific results were provided in 11 articles) were used including 146,830 deaths among 1,526,609 participants. For 24-hour sleep duration, results from 13 articles with 19 results (gender-specific results were provided in 6 articles) were used including 101,641 deaths among 903,727 participants. Detailed characteristics of the included studies are shown in Table S2.

### Quantitative Synthesis

#### Nighttime sleep duration and all-cause mortality

**Figure 1:**
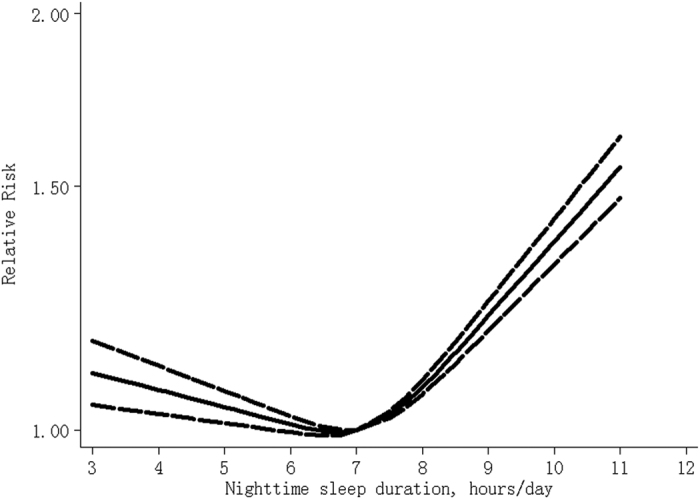
The dose-response analysis between nighttime sleep duration and risk of all-cause mortality. The solid line and the long dash line represent the estimated relative risk and its 95% confidence interval.

Table 1 Compared with 7 hours/day, a U-shaped relationship between nighttime sleep duration and risk of all-cause mortality was found (P_for non-linearity_ <0.001), and the RRs (95% CIs) of all-cause mortality were 1.07 (1.03–1.13), 1.04 (1.01–1.07), 1.01 (1.00–1.02), 1.07 (1.06–1.09), 1.21 (1.18–1.24), 1.37 (1.32–1.42) and 1.55 (1.47–1.63) for 4, 5, 6, 8, 9, 10 and 11 hours/day of nighttime sleep (Fig. 1), respectively. There is low between-study heterogeneity among study-specific trends, defined by the coefficients of the first (I2 = 29%) and second (I2 = 2%) spline transformations of nighttime sleep duration. No publication bias was found (P = 0.57). Sensitivity analysis showed that exclusion of any one study did not influence the relationship (all P values for non-linearity <0.001). Among subjects without cancer and cardiovascular diseases at baseline^21^,^24^,^27^,^28^,^30^,^31^, the risks were 1.23 (1.10–1.37), 1.14 (1.06–1.22), 1.05 (1.02–1.09), 1.04 (1.02–1.05), 1.11 (1.08–1.15), 1.19 (1.14–1.25) and 1.28 (1.21–1.36) for 4, 5, 6, 8, 9, 10 and 11 hours/day, respectively (44,998 death cases among 329,420 participants).

#### 24-hour sleep duration and all-cause mortality

**Figure 2:**
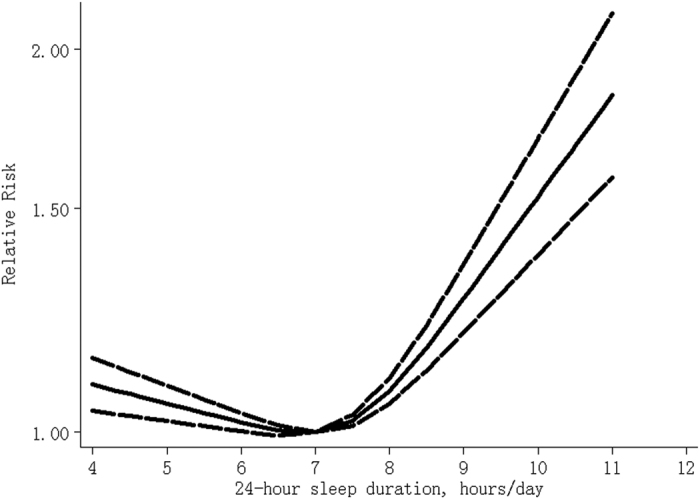
The dose-response analysis between 24-hour sleep duration and risk of all-cause mortality. The solid line and the long dash line represent the estimated relative risk and its 95% confidence interval.

Table 2 Compared with 7 hours/day, a U-shaped relationship between 24-hour sleep duration and risk of all-cause mortality was found (P_for non-linearity_ < 0.001), and the RRs (95% CIs) of all-cause mortality were 1.09 (1.04–1.14), 1.05 (1.02–1.09), 1.02 (1.00–1.03), 1.08 (1.05–1.10), 1.27 (1.20–1.36), 1.53 (1.38–1.70) and 1.84 (1.59–2.13) for 4, 5, 6, 8, 9, 10 and 11 hours/day of 24-hour sleep (Fig. 2), respectively. There is moderate between-study heterogeneity among study-specific trends, defined by the coefficients of the first (I2 = 51%) and second (I2 = 65%) spline transformations of 24-hour sleep duration. No publication bias was found (P = 0.24). Sensitivity analysis showed that exclusion of any one study did not influence the relationship (all P values for non-linearity <0.001). Among subjects without cancer and cardiovascular diseases at baseline^20^,^22^,^25^, the risks were 1.10 (1.04–1.16), 1.06 (1.02–1.10), 1.03 (1.01–1.04), 1.03 (1.01–1.05), 1.09 (1.04–1.15), 1.17 (1.08–1.27) and 1.25 (1.12–1.40) for 4, 5, 6, 8, 9, 10 and 11 hours/day, respectively (26,147 death cases among 351,530 participants).

#### Subgroup analysis and meta-regression (Table S3)

Subgroup and meta-regression were conducted to explore possible sources of between-study heterogeneity. In the analysis of nighttime sleep duration and risk of all-cause mortality, follow-up duration, sleep duration assessment method, mean age, sex, continent where the study were conducted, covariates adjusted for and study quality did not contribute to the heterogeneity significantly (all P values > 0.05). In the analysis of 24-hour sleep duration and all-cause mortality, continent where the study were conducted (P = 0.05) and follow-up duration (P = 0.05) might contribute to the heterogeneity, which may be also caused by chance because of the relatively small number of studies. Other factors did not contribute to the heterogeneity significantly (all P values > 0.05).

## Discussion

Results from this dose-response meta-analysis showed that, compared with 7 hours/day, sleep duration was associated with the risk of all-cause mortality in a U-shaped manner among adults, regardless of nighttime sleep or 24-hour sleep. Overall, study design, population characteristics and other covariates did not influence the relationship substantially.

Five theoretical pathways for the relationship between short sleep duration and mortality have been put forth^32^: (I) short sleep directly causes mortality itself; (II) short sleep may result from variety of social, environmental, and physiological changes that lead to increased mortality risk; (III) short sleep itself causes physiological and social outcomes that may lead to increased mortality; (IV) short sleep is associated with other characteristics causally linked to mortality, such as age; and (V) the possibility of reverse causality is also of concern. Proposed mechanisms for mortality associated with long sleep include^33^: (I) long sleep is linked to increased sleep fragmentation that is associated with a number of negative health outcomes; (II) long sleep is associated with feelings of fatigue and lethargy that may decrease resistance to stress and disease; (III) changes in cytokine levels associated with long sleep increase mortality risk; (IV) long sleepers experience a shorter photoperiod that could increase the risk of death in mammalian species; (V) a lack of physiological challenge with long sleep decrease longevity; (VI) underlying disease processes mediate the relationship between long sleep and mortality. In addition, this meta-analysis suggested that the risk estimations of all-cause mortality associated with longer duration (10 hours and 11 hours) of 24-hour sleep were larger than those of nighttime sleep. The excess risk might be partly attributable to daytime napping that is positively associated with risk of all-cause mortality^34^.

One major concern with the observed associations is that short and long sleep duration might be just markers of poor health status rather than independent predictors. Among the studies included, a U-shaped association was found among subjects with one or more chronic diseases but no association was found among participants without chronic diseases in the 45 and Up Study^22^. However, the U-shaped association was found in both subjects with one or more chronic diseases and subjects without chronic diseases in another study^25^. In addition, no statistical differences in association between sleep duration and all-cause mortality were found in stratified analysis by self-perceived health^13^,^19^, and excluding subjects with prevalent chronic diseases made little or no difference to the overall results^35^,^36^. Reverse causality is of another concern; however, the included studies found that the associations did not changed substantially after excluding early death occurring within the first 1–5 years of follow-up^11^,^13^,^14^,^16^,^24^,^27^,^28^,^30^,^31^,^35^,^37^,^38^,^39^. In addition, in this meta-analysis, the U-shaped relationship was also found among subjects without cardiovascular diseases and cancer at baseline. These findings indicated that preexisting chronic diseases might influence but not fully explain the observed associations. Furthermore, the observed associations remained after adjustment for other covariates. Therefore, these results suggested that both long and short sleep duration should be independent predictors of all-cause mortality.

Other factors influencing the observed associations should also be considered. Although no significant interactions between sleep duration and moderate-to-vigorous physical activity and body mass index on all-cause mortality were found in National Institutes of Health -AARP Diet and Health Study^28^, the U-shaped relationship was mainly observed among subjects with lower levels of physical activity in other studies^20^,^24^, and the interaction with body mass index was also found in another study^20^. In addition, the association of long duration of sleep with all-cause mortality was only observed among subjects taking napping^15^, and the U-shaped relationship was also more pronounced among subjects who napped daily^40^. However, the limited data precluded a more detailed analysis in this meta-analysis.

An earlier review found that both short and long duration of sleep are significant predictors of death^10^. Since the review was published, 19 population-based prospective cohort studies^11^,^12^,^13^,^14^,^15^,^16^,^17^,^18^,^19^,^20^,^21^,^22^,^23^,^24^,^25^,^26^,^27^,^28^,^29^ were also included in this meta-analysis, making it possible to describe the dose-response relationship of sleep duration with risk of all-cause mortality, calculate the risk of all-cause mortality associated with specific duration of sleep, and assess whether other factors influence the association of sleep duration with all-cause mortality.

Other limitations should also be of noteworthy. First, sleep duration was self-reported and, therefore, might be subject to error and misclassification, and participants might also change their sleep pattern during follow-up. Second, while we extracted RRs that reflected the greatest degree of control for potential confounders, the extent to which they were adjusted for and potential residual confounding by other unmeasured factors^41^,^42^,^43^,^44^,^45^ should also be considered. In addition, adjustment for potential factors that may be on the biological pathway between sleep duration and mortality risk would result in over adjustment. However, this meta-analysis showed that covariates adjusted for in the original studies did not influence the observed associations substantially. Third, publication bias could be of concern because small studies with null results tend not to be published; however, no publication bias was detected. In spite of the limitations, results from prospective cohort studies are still the best evidence available to assess the longitudinal effect of sleep duration on mortality.

In summary, results from this meta-analysis showed that both short and long sleep duration were independent predictors of all-cause mortality, and 7 hours/day of sleep should be recommended to prevent premature death among adults.

## Materials and Methods

### Literature search and selection

We performed a literature search up to March 2015 using the databases of Pubmed and Embase, using the following search terms *((((((prospective) OR cohort) OR longitudinal) OR follow-up)) AND ((death) OR mortality)) AND sleep)*, without restrictions. Moreover, we reviewed the reference lists from retrieved articles to search for further relevant studies. There is no protocol for this meta-analysis.

Two investigators (X.S. and Y.W.) independently reviewed all identified studies, and studies were included if they met the following criteria: (1) a prospective design; (2) the exposure of interest was sleep duration; (3) the outcome of interest was all-cause mortality; (4) relative risk (RR) with 95% confidence interval (CI) for 3 or more categories of sleep duration was provided; (5) the study was conducted among adults. If data were duplicated in more than one study, we included the study with the longest follow-up duration.

### Data extraction

The following data were extracted from each study by two investigators (X.S. and Y.W.): publication year, the first author’s last name, country where the study was performed, follow-up duration, total number of participants and death cases, health status of participants at baseline, sleep duration assessment method, variables adjusted for in the analysis, the numbers of death cases and participants (or person-years) and RR estimates with corresponding 95% CI for the each categories of sleep duration. Information was also gained with a request to the corresponding author of original studies^17^,^19^. We extracted RRs that reflected the greatest degree of control for potential confounders.

### Statistical analysis

The trend from the correlated log RR estimates across levels of sleep duration was computed using a two-stage random-effects dose–response meta-analysis^46^, taking into account the between-study heterogeneity. In the first stage, the generalised least-square regression was used to estimate a restricted cubic spline model with three knots at the 25th, 50th and 75th percentiles of the levels of sleep duration. Then a multivariate random-effects meta-analysis was adopted to combine the study-specific estimates using the restricted maximum likelihood method. A P value for non-linearity was calculated by testing the null hypothesis that the coefficient of the second spline is equal to 0. This dose-response meta-analysis requires that the number of death cases and participants (or person-years) and RR estimates with corresponding 95% CI for the each categories of sleep duration should be available. The mean level of sleep duration in each category was assigned to the corresponding RR for every study. If the upper boundary of the highest category was not provided, we assumed that the boundary had the same amplitude as the adjacent category. For studies^15^,^20^,^25^,^26^,^39^,^40^,^47^,^48^ that did not report the numbers of death cases for each categories of sleep duration, these numbers were inferred based on total numbers of cases and the reported crude risk estimates or risk estimates that were adjusted for least number of covariates^49^.

Between-study heterogeneity was assessed with I2, and the values of 25%, 50% and 75% represent low, moderate and high heterogeneity^50^, respectively. Subgroup analysis and meta-regression were conducted to explore potential sources of heterogeneity and perform comparisons between groups, and a permutation test of 1000 was used to obtain P values from meta-regression to control spurious findings^51^. Publication bias was evaluated using Egger test. A sensitivity analysis was performed with one study removed at a time to assess whether the results could have been affected markedly by a single study. Study quality was assessed using the 9-star Newcastle-Ottawa scale (http://www.ohri.ca/programs/clinical_epidemiology/oxford.asp, accessed 12/21/2015). All statistical analyses were performed with STATA version 12.0 (Stata Corporation, College Station, TX, USA). All reported probabilities (P-values) were two-sided with P < 0.05 considered statistically significant.

## Additional Information

**How to cite this article**: Shen, X. *et al.* Nighttime sleep duration, 24-hour sleep duration and risk of all-cause mortality among adults: a meta-analysis of prospective cohort studies. *Sci. Rep.* **6**, 21480; doi: 10.1038/srep21480 (2016).

## Supplementary Material

Supplementary Information

## Figures and Tables

**Table 1:** Pooled measures on the relation of nighttime sleep duration and risk of all-cause mortality.

	RR (95% CI) by hours of nighttime sleep per day									
	N^a^	Deaths	4 hours	5 hours	6 hours	7 hours	8 hours	9 hours	10 hours	11 hours
Overall	36	146,830	1.07 (1.03–1.13)	1.04 (1.01–1.07)	1.01 (1.00–1.02)	1.00	1.07 (1.06–1.09)	1.21 (1.18–1.24)	1.37 (1.32–1.42)	1.55 (1.47–1.63)
Subjects without cardiovascular diseases and cancer at baseline	10	44,998	1.23 (1.10–1.37)	1.14 (1.06–1.22)	1.05 (1.02–1.09)	1.00	1.04 (1.02–1.05)	1.11 (1.08–1.15)	1.19 (1.14–1.25)	1.28 (1.21–1.36)
Country										
Asia	18	21,640	1.07 (0.94–1.22)	1.05 (0.95–1.16)	1.00 (0.97–1.04)	1.00	1.11 (1.08–1.13)	1.36 (1.28–1.43)	1.70 (1.55–1.85)	2.12 (1.87–2.40)
Europe	9	20,186	1.31 (1.15–1.49)	1.18 (1.08–1.29)	1.07 (1.02–1.12)	1.00	1.05 (1.00–1.11)	1.15 (1.03–1.28)	1.26 (1.07–1.48)	—
USA	8	90,732	1.11 (1.07–1.14)	1.06 (1.04–1.09)	1.02 (1.01–1.03)	1.00	1.02 (1.01–1.03)	1.07 (1.05–1.19)	1.11 (1.09–1.14)	1.16 (1.12–1.20)
Sex										
Men	12	39,440	1.12 (1.02–1.21)	1.07 (1.01–1.13)	1.03 (1.00–1.06)	1.00	1.04 (1.02–1.06)	1.14 (1.10–1.18)	1.25 (1.18–1.31)	1.37 (1.28–1.47)
Women	12	70,175	1.07 (1.02–1.12)	1.04 (1.01–1.07)	1.01 (1.00–1.03)	1.00	1.06 (1.05–1.07)	1.18 (1.15–1.21)	1.33 (1.28–1.38)	1.49 (1.41–1.57)
Both	12	37215	1.07 (0.97–1.18)	1.04 (0.97–1.11)	1.00 (0.97–1.04)	1.00	1.09 (1.05–1.12)	1.20 (1.13–1.28)	1.33 (1.21–1.46)	—
Mean age										
>60 years	19	45,824	1.04 (0.96–1.13)	1.02 (0.96–1.08)	1.00 (0.97–1.03)	1.00	1.11 (1.08–1.13)	1.36 (1.30–1.44)	1.71 (1.57–1.87)	2.14 (1.90–2.42)
≤60 years	17	101,006	1.11 (1.07–1.15)	1.07 (1.04–1.09)	1.02 (1.01–1.04)	1.00	1.05 (1.04–1.06)	1.15 (1.12–1.17)	1.25 (1.22–1.29)	1.37 (1.31–1.43)
Follow-up duration										
≥10 years	20	54,615	1.15 (1.07–1.23)	1.09 (1.03–1.14)	1.03 (1.00–1.05)	1.00	1.09 (1.07–1.11)	1.25 (1.21–1.29)	1.44 (1.36–1.52)	1.66 (1.54–1.78)
<10 years	16	92,215	1.00 (0.95–1.06)	1.00 (0.96–1.03)	0.99 (0.97–1.01)	1.00	1.09 (1.06–1.11)	1.24 (1.18–1.29)	1.42 (1.33–1.52)	1.64 (1.49–1.79)
Sleep assessment										
Self- administered	21	134,427	1.11 (1.06–1.16)	1.06 (1.03–1.10)	1.02 (1.00–1.04)	1.00	1.05 (1.04–1.06)	1.14 (1.13–1.16)	1.26 (1.23–1.29)	1.38 (1.34–1.42)
Interviewer- administered	15	12,403	1.09 (0.97–1.21)	1.05 (0.98–1.13)	1.02 (0.98–1.05)	1.00	1.07 (1.04–1.11)	1.21 (1.12–1.31)	1.37 (1.22–1.54)	—
Adjusted for … Education/socioeconomic status										
Yes	27	143,711	1.06 (1.02–1.11)	1.03 (1.01–1.06)	1.01 (0.99–1.02)	1.00	1.09 (1.08–1.11)	1.25 (1.21–1.28)	1.43 (1.37–1.49)	1.64 (1.55–1.74)
No	9	3,119	1.15 (0.95–1.38)	1.09 (0.96–1.23)	1.04 (0.97–1.10)	1.00	1.03 (0.99–1.07)	1.11 (1.02–1.20)	1.20 (1.06–1.36)	—
Smoking										
Yes	28	143,178	1.07 (1.02–1.12)	1.04 (1.01–1.07)	1.01 (0.99–1.02)	1.00	1.10 (1.08–1.11)	1.26 (1.22–1.30)	1.45 (1.38–1.51)	1.66 (1.57–1.77)
No	8	3,652	1.28 (1.03–1.60)	1.17 (1.01–1.36)	1.07 (1.00–1.16)	1.00	1.02 (0.97–1.06)	1.09 (0.99–1.20)	1.18 (1.02–1.38)	—
Alcohol										
Yes	24	56,831	1.08 (1.00–1.16)	1.04 (0.99–1.10)	1.01 (0.98–1.03)	1.00	1.11 (1.09–1.13)	1.29 (1.24–1.34)	1.51 (1.42–1.60)	1.76 (1.63–1.91)
No	12	89,999	1.12 (.08–1.16)	1.07 (1.04–1.09)	1.02 (1.01–1.03)	1.00	1.02 (1.01–1.03)	1.08 (1.06–1.10)	1.14 (1.11–1.17)	1.20 (1.16–1.24)
Physical activity										
Yes	25	142,638	1.07 (1.03–1.10)	1.04 (1.02–1.06)	1.01 (1.00–1.02)	1.00	1.09 (1.08–1.10)	1.25 (1.21–1.28)	1.42 (1.37–1.48)	1.63 (1.54–1.72)
No	11	4,192	1.27 (1.01–1.59)	1.17 (1.00–1.35)	1.07 (0.99–1.16)	1.00	1.01 (0.95–1.07)	1.10 (0.98–1.24)	1.22 (1.02–1.46)	—
Health status^b^										
Yes	10	105,645	1.06 (1.02–1.10)	1.02 (1.00–1.05)	0.99 (0.98–1.00)	1.00	1.09 (1.07–1.11)	1.23 (1.18–1.28)	1.39 (1.31–1.47)	1.57 (1.46–1.69)
No	26	41,195	1.11 (1.03–1.21)	1.07 (1.01–1.13)	1.03 (1.00–1.05)	1.00	1.06 (1.04–1.08)	1.20 (1.15–1.26)	1.38 (1.29–1.48)	1.59 (1.44–1.75)
Blood pressure										
Yes	22	112,841	1.06 (1.02–1.11)	1.03 (1.01–1.06)	1.01 (1.00–1.02)	1.00	1.06 (1.04–1.08)	1.18 (1.14–1.21)	1.31 (1.25–1.38)	1.47 (1.38–1.57)
No	14	33,989	1.13 (1.00–1.28)	1.08 (0.99–1.17)	1.03 (0.99–1.07)	1.00	1.07 (1.04–1.10)	1.19 (1.13–1.26)	1.33 (1.23–1.45)	—
Body mass index										
Yes	26	142,963	1.06 (1.01–1.11)	1.03 (1.00–1.06)	1.00 (0.99–1.02)	1.00	1.09 (1.08–1.11)	1.25 (1.22–1.29)	1.44 (1.38–1.50)	1.65 (1.56–1.74)
No	10	3,867	1.27 (1.06–1.52)	1.16 (1.03–1.31)	1.07 (1.00–1.13)	1.00	1.07 (1.02–1.12)	1.30 (1.13–1.50)	1.62 (1.27–2.06)	—
Preexisting chronic diseases										
Yes^c^	32	145,917	1.07 (1.03–1.12)	1.04 (1.01–1.07)	1.01 (0.99–1.02)	1.00	1.09 (1.08–1.10)	1.24 (1.21–1.27)	1.41 (1.37–1.46)	1.61 (1.54–1.69)
No	4	913	—	1.03 (0.82–1.29)	1.00 (0.88–1.12)	1.00	1.16 (1.07–1.27)	1.44 (1.23–1.69)	—	—
Sleep disorders/sleeping pills										
Yes^d^	13	91,999	1.11 (1.06–1.15)	1.07 (1.04–1.10)	1.03 (1.01–1.04)	1.00	1.03 (1.01–1.05)	1.09 (1.05–1.12)	1.15 (1.09–1.21)	1.21 (1.13–1.30)
No	23	54,831	1.11 (1.03–1.21)	1.07 (1.01–1.13)	1.02 (1.00–1.05)	1.00	1.06 (1.04–1.07)	1.19 (1.15–1.22)	1.33 (1.27–1.40)	1.50 (1.41–1.60)
Depression/mental health										
Yes	15	25,634	1.02 (0.95–1.10)	1.01 (0.96–1.06)	1.00 (0.97–1.02)	1.00	1.11 (1.09–1.14)	1.38 (1.29–1.47)	1.74 (1.56–1.92)	2.18 (1.89–2.52)
No	21	121,196	1.14 (1.06–1.22)	1.07 (1.03–1.13)	1.02 (0.99–1.04)	1.00	1.11 (1.08–1.13)	1.29 (1.24–1.35)	1.51 (1.42–1.61)	1.77 (1.63–1.93)
Study quality										
7–8 stars	13	50,899	1.17 (1.08–1.26)	1.10 (1.04–1.16)	1.03 (1.01–1.06)	1.00	1.07 (1.05–1.09)	1.18 (1.14–1.22)	1.29 (1.23–1.36)	1.42 (1.33–1.51)
4–6 stars	23	95,931	1.02 (0.97–1.07)	1.01 (0.97–1.04)	0.99 (0.98–1.01)	1.00	1.10 (1.08–1.12)	1.32 (1.27–1.38)	1.62 (1.51–1.74)	1.98 (1.79–2.18)

^a^N: number of studies (gender specific studies).

^b^Health status (self-reported or physical functioning).

^c^Includes studies with subjects of no chronic diseases (cardiovascular diseases and cancer) at baseline.

^d^Includes studies with subjects who did not take sleeping pills at baseline.

**Table 2:** Pooled measures on the relation of 24-hour sleep duration and risk of all-cause mortality.

	RR (95% CI) by hours of 24-hour sleep per day									
	N^a^	Deaths	4 hours	5 hours	6 hours	7 hours	8 hours	9 hours	10 hours	11 hours
Overall	19	101,641	1.09 (1.04–1.14)	1.05 (1.02–1.09)	1.02 (1.00–1.03)	1.00	1.08 (1.05–1.10)	1.27 (1.20–1.36)	1.53 (1.38–1.70)	1.84 (1.59–2.13)
Subjects without cardiovascular diseases and cancer at baseline	4	26,147	1.10 (1.04–1.16)	1.06 (1.02–1.10)	1.03 (1.01–1.04)	1.00	1.03 (1.01–1.05)	1.09 (1.04–1.15)	1.17 (1.08–1.27)	1.25 (1.12–1.40)
Country^b^										
Asia	6	14,707	1.01 (0.92–1.11)	1.00 (0.94–1.07)	1.00 (0.96–1.03)	1.00	1.05 (1.03–1.08)	1.15 (1.08–1.22)	1.24 (1.12–1.36)	1.37 (1.20–1.57)
Europe	7	7,386	—	1.18 (0.97–1.43)	1.08 (0.98–1.19)	1.00	1.01 (0.96–1.06)	1.12 (0.97–1.28)	1.22 (0.97–1.47)	1.37 (0.97–1.95)
USA	5	70,766	1.07 (1.03–1.11)	1.04 (1.02–1.07)	1.02 (1.00–1.03)	1.00	1.01 (1.00–1.03)	1.03 (1.01–1.06)	1.08 (1.02–1.14)	—
Sex										
Men	6	17,462	1.16 (0.99–1.35)	1.10 (0.99–1.22)	1.05 (0.99–1.10)	1.00	1.09 (1.03–1.15)	1.34 (1.12–1.61)	1.66 (1.21–2.27)	2.06 (1.32–3.21)
Women	8	21,046	1.11 (1.01–1.23)	1.07 (1.00–1.14)	1.02 (0.99–1.06)	1.00	1.09 (1.04–1.15)	1.34 (1.17–1.53)	1.67 (1.34–2.09)	2.09 (1.53–2.85)
Both	5	63,133	1.06 (1.00–1.13)	1.04 (1.00–1.08)	1.01 (0.99–1.03)	1.00	1.04 (1.02–1.06)	1.14 (1.10–1.18)	1.24 (1.17–1.31)	1.38 (1.27–1.49)
Mean age										
>60 years	11	84,110	1.06 (1.01–1.11)	1.04 (1.00–1.07)	1.01 (1.00–1.03)	1.00	1.06 (1.04–1.08)	1.21 (1.16–1.26)	1.39 (1.30–1.49)	1.60 (1.45–1.76)
≤60 years	8	17,531	1.19 (1.06–1.34)	1.12 (1.04–1.21)	1.06 (1.02–1.10)	1.00	1.03 (1.00–1.06)	1.15 (1.07–1.24)	1.30 (1.14–1.48)	1.46 (1.22–1.75)
Follow-up duration										
≥10 years	11	83,959	1.12 (1.05–1.19)	1.07 (1.03–1.12)	1.03 (1.01–1.05)	1.00	1.03 (1.02–1.05)	1.13 (1.08–1.18)	1.23 (1.14–1.33)	1.35 (1.21–1.50)
<10 years	8	17,682	1.07 (1.00–1.16)	1.04 (0.99–1.10)	1.01 (0.99–1.04)	1.00	1.07 (1.03–1.11)	1.28 (1.16–1.42)	1.61 (1.33–1.89)	1.95 (1.52–2.48)
Sleep assessment										
Self- administered	15	95,560	1.09 (1.03–1.15)	1.05 (1.01–1.09)	1.02 (1.00–1.04)	1.00	1.07 (1.04–1.09)	1.24 (1.16–1.32)	1.46 (1.31–1.63)	1.72 (1.47–2.00)
Interviewer- administered	4	6,081	1.11 (1.01–1.22)	1.06 (1.00–1.12)	1.02 (0.99–1.05)	1.00	1.11 (1.05–1.17)	1.40 (1.22–1.60)	1.86 (1.25–2.47)	2.32 (1.70–3.18)
Adjusted for … Education/socioeconomic status										
Yes	17	94,310	1.11 (1.06–1.18)	1.07 (1.03–1.11)	1.03 (1.01–1.04)	1.00	1.07 (1.05–1.09)	1.26 (1.18–1.34)	1.50 (1.35–1.67)	1.79 (1.54–2.08)
No	2	7,331	1.02 (0.91–1.14)	1.01 (0.94–1.09)	1.00 (0.97–1.04)	1.00	1.04 (0.99–1.09)	1.12 (1.01–1.23)	1.24 (1.05–1.47)	—
Smoking										
Yes	18	101,417	1.09 (1.04–1.15)	1.06 (1.02–1.09)	1.02 (1.00–1.04)	1.00	1.08 (1.05–1.10)	1.27 (1.19–1.35)	1.52 (1.37–1.70)	1.83 (1.57–2.13)
No	1	224	—							
Alcohol										
Yes	18	101,417	1.09 (1.04–1.15)	1.06 (1.02–1.09)	1.02 (1.00–1.04)	1.00	1.08 (1.05–1.10)	1.27 (1.19–1.35)	1.52 (1.37–1.70)	1.83 (1.57–2.13)
No	1	224	—							
Physical activity										
Yes	17	99,837	1.09 (1.04–1.14)	1.05 (1.02–1.09)	1.02 (1.00–1.03)	1.00	1.08 (1.05–1.10)	1.27 (1.19–1.36)	1.53 (1.36–1.71)	1.83 (1.56–2.15)
No	2	1,804	1.01 (0.68–1.51)	1.00 (0.78–1.22)	0.99 (0.87–1.13)	1.00	1.12 (0.97–1.29)	1.40 (1.03–1.91)	1.83 (1.10–2.81)	2.27 (1.18–4.37)
Health status^c^										
Yes	10	65,418	—	1.03 (0.98–1.08)	1.01 (0.99–1.04)	1.00	1.04 (1.02–1.06)	1.14 (1.09–1.19)	1.27 (1.18–1.38)	1.41 (1.27–1.57)
No	9	36,223	1.14 (1.06–1.22)	1.08 (1.03–1.14)	1.03 (1.01–1.06)	1.00	1.04 (1.02–1.07)	1.15 (1.09–1.22)	1.27 (1.17–1.39)	1.41 (1.25–1.59)
Blood pressure										
Yes	12	40,558	1.07 (1.01–1.14)	1.04 (1.00–1.08)	1.01 (1.00–1.03)	1.00	1.07 (1.04–1.10)	1.24 (1.16–1.33)	1.46 (1.30–1.64)	1.72 (1.46–2.03)
No	7	61,083	—	1.09 (1.01–1.17)	1.03 (0.99–1.07)	1.00	1.10 (1.05–1.15)	1.38 (1.20–1.57)	1.78 (1.39–2.20)	2.25 (1.62–3.12)
Body mass index										
Yes	18	101,417	1.09 (1.04–1.15)	1.06 (1.02–1.09)	1.02 (1.00–1.04)	1.00	1.08 (1.05–1.10)	1.27 (1.19v1.35)	1.52 (1.37–1.70)	1.83 (1.57–2.13)
No	1	224	—							
Preexisting chronic diseases										
Yes^d^	14	95,152	1.08 (1.04v1.12)	1.04 (1.02–1.07)	1.01 (1.00–1.03)	1.00	1.05 (1.04v1.07)	1.16 (1.12–1.20)	1.29 (1.21–1.37)	1.44 (1.32–1.56)
No	5	6,489	—	1.16 (0.90–1.50)	1.06 (0.93–1.21)	1.00	1.06 (0.88–1.28)	1.16 (0.81–1.68)	—	—
Sleep disorders										
Yes	6	8,631	0.97 (0.84–1.13)	0.98 (0.89–1.08)	0.98 (0.94–1.03)	1.00	1.08 (1.03–1.13)	1.23 (1.10–1.37)	1.42 (1.19–1.70)	1.64 (1.27v2.10)
No	13	93,010	1.13 (1.07–1.19)	1.08 (1.04–1.12)	1.03 (1.01–1.05)	1.00	1.05 (1.03–1.07)	1.17 (1.11–1.23)	1.31 (1.21–1.43)	1.47 (1.31–1.65)
Depression/mental health										
Yes	5	16,675	0.99 (0.92–1.07)	0.99 (0.95–1.04)	1.00 (0.97–1.02)	1.00	1.02 (1.00–1.04)	1.11 (1.06–1.17)	1.23 (1.12–1.35)	1.36 (1.19–1.56)
No	14	84,966	1.12 (1.06–1.18)	1.07 (1.03–1.11)	1.02 (1.00–1.04)	1.00	1.12 (1.08–1.17)	1.40 (1.26–1.57)	1.76 (1.47–2.10)	2.21 (1.73–2.83)
Study quality										
7–8 stars	10	86,848	1.12 (1.06–1.80)	1.07 (1.03–1.11)	1.03 (1.01–1.05)	1.00	1.06 (1.04–1.08)	1.20 (1.14–1.26)	1.36 (1.25–1.47)	1.54 (1.38–1.73)
4–6 stars	9	14,793	—	1.03 (0.95–1.12)	1.01 (0.97–1.06)	1.00	1.04 (0.99–1.10)	1.17 (1.02–1.33)	1.32 (1.04–1.67)	1.49 (1.08–2.07)

^a^N: number of studies (gender specific studies).

^b^there is a study conduced in Australia.

^c^health status (self-reported health status or physical functioning).

^d^includes the studies with subjects of no chronic diseases (cardiovascular diseases and cancer) at baseline.
