# Supplementary material for: Nighttime sleep duration, 24-hour sleep duration and risk of all-cause mortality among adults: a meta-analysis of prospective cohort studies
Source: Sci Rep. 2016 Feb 22;6:21480. doi: 10.1038/srep21480 (PMC4761879; doi:10.1038/srep21480)
Supplement: Supplementary Information [file srep21480-s1.pdf]

Title: Nighttime sleep duration, 24-hour sleep duration and risk of all-cause mortality among adults: a meta-analysis of prospective cohort studies

Authors: Xiaoli Shen, Yili Wu, Dongfeng Zhang

Corresponding author: Dongfeng Zhang (zhangdf1961@126.com)

Table S1 The PRISMA checklist

Section/topic	#	Checklist item	Reported on page #
**TITLE**			
Title	1	Identify the report as a systematic review, meta-analysis, or both.	1
**ABSTRACT**			
Structured summary	2	Provide a structured summary including, as applicable: background; objectives; data sources; study eligibility criteria, participants, and interventions; study appraisal and synthesis methods; results; limitations; conclusions and implications of key findings; systematic review registration number.	2
**INTRODUCTION**			
Rationale	3	Describe the rationale for the review in the context of what is already known.	3
Objectives	4	Provide an explicit statement of questions being addressed with reference to participants, interventions, comparisons, outcomes, and study design (PICOS).	3
**METHODS**			
Protocol and registration	5	Indicate if a review protocol exists, if and where it can be accessed (e.g., Web address), and, if available, provide registration information including registration number.	9
Eligibility criteria	6	Specify study characteristics (e.g., PICOS, length of follow-up) and report characteristics (e.g., years considered, language, publication status) used as criteria for eligibility, giving rationale.	9-10
Information sources	7	Describe all information sources (e.g., databases with dates of coverage, contact with study authors to identify additional studies) in the search and date last searched.	10
Search	8	Present full electronic search strategy for at least one database, including any limits used, such that it could be repeated.	9
Study selection	9	State the process for selecting studies (i.e., screening, eligibility, included in systematic review, and, if applicable, included in the meta-analysis).	9-10

Data collection process	10	Describe method of data extraction from reports (e.g., piloted forms, independently, in duplicate) and any processes for obtaining and confirming data from investigators.	10
Data items	11	List and define all variables for which data were sought (e.g., PICOS, funding sources) and any assumptions and simplifications made.	10
Risk of bias in individual studies	12	Describe methods used for assessing risk of bias of individual studies (including specification of whether this was done at the study or outcome level), and how this information is to be used in any data synthesis.	11
Summary measures	13	State the principal summary measures (e.g., risk ratio, difference in means).	10-11
Synthesis of results	14	Describe the methods of handling data and combining results of studies, if done, including measures of consistency (e.g., I^2) for each meta-analysis.	10-11
Risk of bias across studies	15	Specify any assessment of risk of bias that may affect the cumulative evidence (e.g., publication bias, selective reporting within studies).	11
Additional analyses	16	Describe methods of additional analyses (e.g., sensitivity or subgroup analyses, meta-regression), if done, indicating which were pre-specified.	10-11
**RESULTS**			
Study selection	17	Give numbers of studies screened, assessed for eligibility, and included in the review, with reasons for exclusions at each stage, ideally with a flow diagram.	3-4
Study characteristics	18	For each study, present characteristics for which data were extracted (e.g., study size, PICOS, follow-up period) and provide the citations.	3-4
Risk of bias within studies	19	Present data on risk of bias of each study and, if available, any outcome level assessment (see item 12).	4
Results of individual studies	20	For all outcomes considered (benefits or harms), present, for each study: (a) simple summary data for each intervention group (b) effect estimates and confidence intervals, ideally with a forest plot.	4-5
Synthesis of results	21	Present results of each meta-analysis done, including confidence intervals and measures of consistency.	4-5
Risk of bias across studies	22	Present results of any assessment of risk of bias across studies (see Item 15).	4-5
Additional analysis	23	Give results of additional analyses, if done (e.g., sensitivity or subgroup analyses, meta-regression [see Item 16]).	5-6

**DISCUSSION**			
Summary of evidence	24	Summarize the main findings including the strength of evidence for each main outcome; consider their relevance to key groups (e.g., healthcare providers, users, and policy makers).	6-8
Limitations	25	Discuss limitations at study and outcome level (e.g., risk of bias), and at review-level (e.g., incomplete retrieval of identified research, reporting bias).	8-9
Conclusions	26	Provide a general interpretation of the results in the context of other evidence, and implications for future research.	9
**FUNDING**			
Funding	27	Describe sources of funding for the systematic review and other support (e.g., supply of data); role of funders for the systematic review.	12

From: Moher D, Liberati A, Tetzlaff J, Altman DG, The PRISMA Group (2009). Preferred Reporting Items for Systematic Reviews and Meta-Analyses: The PRISMA Statement. PLoS Med 6(6): e1000097. doi:10.1371/journal.pmed1000097

For more information, visit: **www.prisma-statement.org**.

Table S2 characteristics of the studies included in this meta-analysis

Author, year, cohort name, country	Exposure	Mean age (years), sex	Subjects (cases)	Follow-up (years)	RR (95% CI) for each categories of sleep duration	Covariates adjusted
Tsubono et al. 1993, none, Japan[1]	Nighttime sleep	61.4, both	4318 (207)	4	≤6: 1.26 (0.81-1.97)<br>7-8: 1.00<br>≥9: 1.58 (1.16-2.15)	Age and sex
Ruigómez et al. 1995, HISB, Spain[2]	24-hour sleep	73.9, both	1219 (224)	5	<7: 0.83 (0.56-1.23)<br>7-9: 1.00<br>>9: 1.37 (0.89-2.11)	Age, sex, education level and self-perceived health status
Gale et al. 1998, none, Britain[3]	Nighttime sleep	74.0, both	1229 (1158)	23	≤7: 1.0 (0.7-1.4)<br>8: 0.8 (0.7-1.0)<br>9: 1.0<br>10: 1.2 (1.0-1.4)<br>11: 1.3 (1.0-1.7)<br>≥12: 1.7 (1.2-2.5)	Age, sex, geriatrician's diagnoses of illness, social class, SBP, and BMI
Kojima, et al. 2000, none, Japan[4]	Nighttime sleep	M: 46.9<br>F: 47.7	M: 2438 (149)<br>W: 2884 (109)	11.9	M: W:<br>≤6.9: 1.93 (1.12-3.35) 0.90 (0.50-1.61)<br>7.0-8.9: 1.00 1.00<br>9.0-9.9: 1.15 (0.74-1.77) 1.07 (0.58-1.95)<br>≥10.0: 1.77 (0.88-3.54) 0.40 (0.06-2.92)	Age, present and past history of hypertension, cerebrovascular, heart and renal diseases and diabetes, and use of sleeping pills (smoking and drinking habits only in males)
Heslop et al. 2002, none, UK[5]	24-hour sleep	≤65	M: 6022 (2303)<br>W: 1006 (262)	25.0	M: W:<br><7: 1.00 (0.89-1.12) 0.98 (0.70-1.37)<br>7-8: 1.00 1.00<br>>8: 0.81 (0.67-0.97) 1.20 (0.71-2.05)	Age, marital status, current own social class, DBP, blood cholesterol level, BMI, FEV1, hours of recreational exercise per week, units of alcohol consumed each week, number of cigarettes smoked each day

Kripke et al. 2002, CPSII, USA[6]	Nighttime sleep	M: 58<br>W: 57	M: 480841 (24523)<br>W: 636095 (59793)	6.0	W: M:<br>3: 1.33 (1.08-1.64) 1.19 (0.96-1.47)<br>4: 1.11 (1.01-1.22) 1.17 (1.06-1.28)<br>5: 1.07 (1.01-1.13) 1.11 (1.05-1.18)<br>6: 1.07 (1.03-1.11) 1.08 (1.04-1.11)<br>7: 1.00 1.00<br>8: 1.13 (1.09-1.16) 1.12 (1.09-1.15)<br>9: 1.23 (1.17-1.28) 1.17 (1.13-1.21)<br>≥10: 1.41 (1.34-1.50) 1.34 (1.28-1.40)	Age, race education, occupation, marital status, exercise level, smoking at intake, years of smoking, churchgoing, fat in diet, fiber in diet, insomnia frequency, health, BMI, leg pain, history of heart disease, hypertension, cancer, diabetes, stroke, respiratory disease, kidney disease and medications
Mallon et al. 2002, none, Sweden[7]	Nighttime sleep	45-65 (56)	M: 906 (165)<br>W: 964 (101)	12	M: W:<br><6: 1.1 (0.6-7.0) 1.0 (0.6-1.8)<br>6-8: 1.0 1.00<br>>8: 2.0 (1.2-3.2) 1.3 (0.6-2.6)	Age
Burazeri et al. 2003, KYCHS, Israel[8]	Nighttime sleep	M: 64<br>W: 63	M: 841 (198)<br>W: 1001 (205)	9-11	M: W:<br><6: 1.00 1.00<br>6-8: 1.25 (0.83-1.87) 0.80 (0.54-1.17)<br>>8: 1.91 (1.16-3.13) 1.08 (0.70-1.66)	Age, social class, country of origin, education, health status, PH of diabetes, CHD, stroke, CHF, smoking, alcohol consumption, physical activity, blood pressure, BMI, glucose, thiocyanate, creatinine, albumin, TC, HDL, homocysteine and siesta (for night sleep)
	24-hour sleep				M: W:<br><6: 1.00 1.00<br>6-8: 1.41 (0.83-2.39) 0.64 (0.42-0.97)<br>>8: 2.13 (1.23-3.71) 0.80 (0.51-1.24)	
Amagai, et al. 2004, JMSCS, Japan[9]	Nighttime sleep	M: 55.0<br>W: 55.2	M: 4419 (289)<br>W: 6906 (206)	8.2	M: W:<br>≤5.9: 2.4 (1.3-4.2) 0.7 (0.2-2.3)<br>6.0-6.9 1.1 (0.7-1.8) 1.3 (0.8-2.1)<br>7.0-7.9: 1.0 1.0<br>8.0-8.9: 0.9 (0.6-1.2) 1.1 (0.8-1.6)<br>≥9.0: 1.1 (0.8-1.6) 1.5 (1.0-2.4)	Age, SBP, total cholesterol, BMI, smoking habits, alcohol drinking habits, education, and marital status
Patel, et al, 2004, NHS,	24-hour sleep	W: 53.4	82969 (5409)	14.0	≤5: 1.08 (0.96-1.22)<br>6: 0.99 (0.92-1.06)	Age, smoking status, alcohol consumption, physical activity,

USA[10]					7: 1.00<br>8: 1.11 (1.03-1.19)<br>≥9: 1.40 (1.25-1.55)		depression, history of snoring, BMI, history of cancer , cardiovascular disease, hypertension, diabetes and shift-working
Ferrie, 2007, Whitehall II study, UK[11]	Nighttime sleep	44.6, both	9871 (566)	17.1	≤5: 1.24 (0.92-1.67)<br>6: 1.00 (0.82-1.22)<br>7: 1.00<br>8: 1.07 (0.84-1.36)<br>≥9: 1.54 (0.72-3.28)		Age, sex, marital status, employment grade, smoking status, physical activity, alcohol consumption, self-rated health, BMI, SBP, cholesterol, physical illness, modified GHQ score, prevalent CHD
Hubin et al. 2007, Finnish Twin Cohort, Finland[12]	24-hour sleep	40.7	M: 9529 (1850)<br>W: 10265 (1850)	22.0	M:<br><7: 1.26 (1.11-1.43)<br>7-8: 1.00<br>>8: 1.24 (1.09-1.41)	W:<br>1.21 (1.05-1.40)<br>1.00<br>1.17 (1.03-1.34)	Age, education, marital status, working status, social class, BMI, smoking status, binge drinking, grams of alcohol consumed daily, conditioning physical activity, and life satisfaction
Lan, et al. 2007, SHLSE, Taiwan[13]	Nighttime sleep	M: 71.3<br>W: 71.9	M: 1748 (816)<br>W: 1331 (522)	8.4	M:<br><7: 0.98 (0.76-1.25)<br>7-7.9: 1.00<br>8-8.9: 1.09 (0.89-1.33)<br>9-9.9: 1.14 (0.91-1.42)<br>≥10: 1.51 (1.19-1.92)	W:<br>1.14 (0.77-1.67)<br>1.00<br>1.36 (1.01-1.84)<br>1.86 (1.36-2.53)<br>2.06 (1.50-2.83)	Age, marital status, monthly income, cigarettes smoking, alcohol consumption, BMI, exercise, depression, disease history of heart disease, stroke and cancer, afternoon nap duration
Gangwisch et al. 2008, NHANES I, USA[14]	Nighttime sleep	56.4, both	9789 (1877)	8-10	≤5: 1.17 (0.99-1.39)<br>6: 0.95 (0.81-1.11)<br>7: 1.00<br>8: 1.23 (1.08-1.39)<br>≥9: 1.34 (1.15-1.56)		Age, sex, physical activity, smoking, depression, education, living alone, low income, daytime sleepiness, nighttime awakening, ethnicity, sleeping pill use, body weight, diabetes, hypertension, general health and cancer

Ikehara et al. 2009, JACC, Japan[15]	Nighttime sleep	M: 58.8<br>W: 60.2	41489 (8548)<br>57145 (5992)	14.3	M:<br>≤4: 1.29 (1.02-1.64)<br>5: 1.02 (0.90-1.16)<br>6: 1.08 (1.00-1.16)<br>7: 1.00<br>8: 1.06 (1.00-1.12)<br>9: 1.13 (1.05-1.22)<br>≥10: 1.41 (1.29-1.54)	W:<br>1.28 (1.03-1.60)<br>1.11 (0.98-1.25)<br>1.05 (0.97-1.14)<br>1.00<br>1.16 (1.08-1.24)<br>1.32 (1.20-1.45)<br>1.56 (1.40-1.75)	Age, BMI, history of hypertension and diabetes, alcohol consumption, smoking, education level, hours of exercise, hours of walking, regular employment, perceived mental stress, depressive symptoms and frequency of fresh fish intake
Stone, et al. 2009, SOF, USA[16]	Nighttime sleep<br><br>24-hour sleep	W: 77.0	8101 (1922)	6.9	Nighttime sleep<br><6: 1.02 (0.87-1.19)<br>6-8: 1.00<br>>8: 1.16 (0.97-1.39)	24-hour sleep<br><6: 0.95 (0.76-1.18)<br>6-8: 1.07 (0.94-1.22)<br>8-9: 1.00<br>9-10: 1.28 (1.08-1.52)<br>≥10: 1.58 (1.27-1.95)	Age, BMI, history of at least one medical condition including diabetes mellitus, Parkinson's disease, dementia, chronic obstructive pulmonary disease, non-skin cancer, and osteoarthritis, history of cardiovascular disease, history of hypertension, walks for exercise, alcohol use, smoking status, depression, cognitive impairment, estrogen use, and benzodiazepine use
Suzuki et al. 2009, Shizuoka Study, Japan[17]	Nighttime sleep	74.1	M:<br>6423 (738)<br>W:<br>6178 (336)	6	M:<br>≤5: 1.08 (0.72-1.61)<br>6: 1.05 (0.75-1.47)<br>7: 1.00<br>8: 1.36 (1.04-1.78)<br>9: 1.52 (1.08-2.15)<br>≥10: 1.86 (1.34-2.56)	W:<br>0.71 (0.39-1.29)<br>1.08 (0.67-1.74)<br>1.00<br>1.39 (0.92-2.09)<br>1.15 (0.64-2.09)<br>2.27 (1.37-3.76)	Age, BMI, smoking status, alcohol consumption, the frequency of physical activity, socioeconomic status, mental health, hypertension and diabetes mellitus.
Chien et al. 2010, CSCCC, Taiwan[18]	Nighttime sleep	54.8, both	3430 (901)	15.9	≤5: 1.15 (0.90-1.46)<br>6: 0.97 (0.79-1.21)<br>7: 1.00		Age, sex, BMI, smoking, current alcohol drinking, marital status, education level, occupation, regular

					8: 1.04 (0.86-1.27)<br>≥9: 1.34 (1.08-1.67)		exercise, family history of coronary heart disease, hypertension, diabetes, cholesterol, HDL, triglyceride, glucose, and uric acid level
Mesas, et al. 2010, Spain[19]	24-hour sleep	71.8	1673 (452)<br>2147 (445)	6.8	M:<br>≤6: 1.16 (0.77-1.73)<br>7: 1.00<br>8: 1.52 (1.04-2.20)<br>9: 1.55 (1.04-2.30)<br>≥10: 1.93 (1.32-2.81)	W:<br>1.52 (1.03-2.23)<br>1.00<br>1.21 (0.81-1.88)<br>1.48 (0.99-2.23)<br>1.67 (1.14-2.44)	Age, BMI, educational level, municipality of residence, physical activity, smoking, alcohol consumption, coffee consumption, social links, perceived health, MEC score, depression, SF-36 PCS and MCS scores, IADL limitations, hypertension, ischemic heart disease, stroke, diabetes mellitus, cancer at any site, chronic obstructive pulmonary disease, Parkinson's disease, arousal from sleep at night, and use of anxiolytic medication
Castro-Costa et al. 2011, Bambuı' Cohort Study, Brazil[20]	Nighttime sleep	68.9, both	1512 (440)	9	<6: 1.09 (0.78-1.53)<br>6-7: 0.84 (0.60-1.17)<br>7-8: 1.00<br>8-9: 1.31 (0.97-1.78)<br>≥9: 1.53 (1.12-2.09)		Age, sex, schooling marital status, working status, education, alcohol consumption, coffee consumption, smoking, physical exercises, apnoea, depressive symptoms, cognitive functioning, psychoactive medications, physical functioning, arthritis ascertainment, SBP, HDL cholesterol ratio, diabetes mellitus and BMI
Cohen-Mansfield et al. 2012,	Nighttime sleep	83.4, both	1166 (1108)	20	<7: 0.98 (0.84-1.13)<br>7-9: 1.00		Age, sex, country of origin, education, financial status, and

CALAS, Israel[21]					>9: 1.32 (1.09-1.58)		having children, married, No. of medications, comorbidity, subjective health, ADL limitations, IADL limitations, cognitive difficulties, depressed affect
Chen et al. 2013, Shih-Pai Sleep Study Taiwan[22]	Nighttime sleep	73.8, both	4064 (1004)	9	≤4: 1.00 (0.75-1.33)<br>5: 0.92 (0.74-1.15)<br>6: 0.88 (0.73-1.06)<br>7: 1.00<br>8: 1.26 (1.04-1.53)<br>≥9: 1.66 (1.28-2.17)		Sex, age, living status, marital status, education, BMI, insomnia, excessive daytime sleepiness, pain, smoking, alcohol drinking, snorers, diabetes mellitus, hypertension, cardiovascular disease, stroke, gouty arthritis, depression and hypnotics
Garde et al. 2013, Copenhagen Male Study, Denmark[23]	Nighttime sleep	48.6, M	5249 (2663)	30	<6: 1.06 (0.90-1.25)<br>6-7: 1.00<br>≥8: 0.99 (0.84-1.09)		Age, BMI, SBP, DBP, diabetes, hypertension, physical fitness, alcohol use, smoking, leisure-time physical activity, and social class
Jung et al. 2013, None, USA[24]	Nighttime sleep	M: 74.1<br>W: 73.3	M: 889 (632)<br>W: 1112 (592)	19	M:<br><6: 0.98 (0.67-1.43)<br>6-6.9: 1.12 (0.85-1.48)<br>7-7.9: 1.00<br>8-8.9: 0.98 (0.79-1.22)<br>≥9: 1.09 (0.82-1.45)	W:<br>1.11 (0.77-1.60)<br>1.17 (0.85-1.61)<br>1.00<br>1.19 (0.90-1.57)<br>1.51 (1.05-2.18)	Age, nap duration, Beck Depression Inventory (only in men), education (only in men), exercise (only in men), smoking (only in women), alcohol consumption, and medical history of hypertension, diabetes, coronary heart disease, stroke, cancer, sleep-related medications and postmenopausal estrogen (only in women)
Kakizaki et al. 2013, Ohsaki Cohort Study,	24-hour sleep	60.3, both	49256 (8447)	10.8	≤6: 1.01 (0.93-1.09)<br>7: 1.00<br>8: 1.07 (1.01-1.14)		Age, sex, total caloric intake, BMI, marital status, level of education, job status, history of myocardial

Japan[25]					9: 1.14 (1.06-1.24)<br>≥10: 1.37 (1.27-1.47)		infarction, history of cancer, stroke, hypertension and diabetes mellitus; smoking status, alcohol drinking, time spent walking, perceived mental stress, self-rated health, physical function
Kim et al. 2013, Multiethnic Cohort Study, USA[26]	24-hour sleep	40-75 (66)	M: 61936 (10738)<br>W: 73749 (8597)	12.9	M:<br>≤5: 1.15 (1.06-1.23)<br>6: 1.04 (0.99-1.10)<br>7: 1.00<br>8: 1.07 (1.01-1.12)<br>≥9: 1.19 (1.12-1.27)	W:<br>1.14 (1.06-1.23)<br>1.05 (0.99-1.12)<br>1.00<br>1.02 (0.96-1.08)<br>1.22 (1.13-1.31)	Age, ethnicity, education, marital status, history of hypertension or diabetes at enrollment, alcohol consumption, energy intake, BMI, physical activity, hours spent daily watching television, and smoking history
Li et al. 2013, SAKUCESS, Japan[27]	Nighttime sleep	M: 57.7<br>W: 52.6	M: 4770 (181)<br>W: 7719 (131)	7	M:<br>≤5: 1.44 (0.65-3.19)<br>6: 0.86 (0.50-1.48)<br>7: 1.00<br>8: 1.05 (0.72-1.53)<br>≥9: 1.70 (1.07-2.70)	W:<br>1.01 (0.42-2.39)<br>1.31 (0.78-2.21)<br>1.00<br>1.01 (0.63-1.60)<br>1.85 (1.09-3.13)	Age, BMI, SBP, DBP, smoking status, drinking habits and physical activity
Magee et al. 2013, 45 and Up Study, Australia[28]	24-hour sleep	61.5, both	227815 (8782)	2.8	<6: 1.13 (1.01-1.25)<br>6: 0.99 (0.91-1.06)<br>7: 1.00<br>8: 1.02 (0.96-1.08)<br>9: 1.04 (0.96-1.12)<br>≥10: 1.26 (1.16-1.36)		Age, gender, marital status, private health insurance, smoking status, alcohol consumption, BMI, sufficient physical activity, and baseline health status
Yeo et al. 2013, KMCC, Korea[29]	24-hour sleep	54.9, both	13164 (1580)	9.44	≤5: 1.21 (1.03-1.41)<br>6: 1.10 (0.95-1.27)<br>7: 1.00<br>8: 1.03 (0.89-1.19)		Age, sex, educational attainment, BMI, cigarette smoking, alcohol consumption, past history of hypertension, type 2 diabetes, CVD,

					9: 1.36 (1.11-1.67)<br>≥10: 1.36 (1.07-1.72)		and metabolic syndrome
Bellavia et al. 2014, CSM and SMC, Sweden[30]	Nighttime sleep	60.2, both	70973 (14575)	15	<6: 1.25 (1.13-1.37)<br>6-6.5: 1.10 (1.04-1.17)<br>6.6-7.4: 1.00<br>7.5-8: 1.03 (0.98-1.08)<br>>8: 1.14 (1.05-1.24)		Sex, age, BMI, smoking status and pack-years of smoking, alcohol consumption, educational level and physical activity
Cai et al. 2014, SWMHS, China[31]	24-hour sleep	M: 55.2<br>W: 52.4	M: 44590 (1921)<br>W: 68548 (2356)	M: 6.07<br>W: 7.12	M:<br>4-5: 1.06 (0.90-1.25)<br>6: 1.07 (0.94-1.23)<br>7: 1.00<br>8: 1.13 (1.00-1.28)<br>9: 1.34 (1.10-1.62)<br>≥10: 1.55 (1.29-1.86)	W:<br>1.15 (1.01-1.32)<br>1.06 (0.94-1.20)<br>1.00<br>1.17 (1.04-1.32)<br>1.36 (1.13-1.64)<br>2.11 (1.77-2.52)	Age, education, income, smoking, alcohol consumption, tea consumption, comorbidity score, history of nightshift work, participation in regular exercise, BMI, and WHR
Rod, et al. 2014, Whitehall II study, UK[32]	Nighttime sleep	M: 43.9<br>W: 45.1	M:<br>6114 (538)<br>W:<br>2984 (266)	22	M:<br>≤5: 1.11 (0.73-1.68)<br>6: 1.23 (1.01-1.50)<br>7: 1.00<br>8: 1.18 (0.92-1.50)<br>>9: 1.44 (0.59-3.50)	W:<br>1.21 (0.76-1.91)<br>1.14 (0.86-1.52)<br>1.00<br>0.91 (0.63-1.30)<br>1.48 (0.60-3.65)	Age, employment grade, ethnicity, and marital status
Xiao et al. 2014, NIH-AARP Diet and Health Study, USA[33]	24-hour sleep<br><br>Nighttime sleep	62.5, both	239896 (44100)	14	24-hour sleep<br><5: 1.16 (1.10-1.23)<br>5-6: 1.04 (1.02-1.06)<br>7-8: 1.00<br>≥9: 1.11 (1.06-1.19)	Nighttime sleep<br>1.20 (1.08-1.33)<br>1.05 (1.01-1.09)<br>1.00<br>1.14 (1.05-1.24)	Sex, age, race/ethnicity, marital status, education, self-reported health, smoking, smoking dose, years since quitting smoking, alcohol drinking, MVPA, television viewing, and BMI
Hall et al. 2015, Health ABC, USA[34]	Nighttime sleep	73.6, both	3075 (953)	8.2	<6: 1.06 (0.83-1.34)<br>6: 1.00 (0.82-1.22)<br>7: 1.00<br>8: 1.10 (0.91-1.33)		Age, gender, race, inflammatory markers (TNF-a, IL6, CRP), education, BMI, alcohol, smoking status, physical activity, study site

					>8: 1.23 (0.93-1.63)	Pittsburgh, arthritis, diabetes, depression, CHD, corticosteroid use, anti-inflammatory use
Zuurbier et al. 2015, Rotterdam Study, The Netherlands[35]	Nighttime sleep	62.2, both	1734 (154)	7.3	<6: 1.41 (0.93-2.13)<br>6-7.5: 1.00<br>>7.5: 1.10 (0.74-1.64)	Age, sex, activities of daily living score, current smoking, diabetes, myocardial infarction, stroke, cognitive functioning, depressive symptoms, BMI, sleep medication, napping, and apnea

BMI: body mass index, CALAS: Cross-Sectional and Longitudinal Aging Study, CHD: coronary heart disease, CSCCC: Chin-Shan Community Cardiovascular Cohort study, CSM and SMC: Cohort of Swedish Men and the Swedish Mammography Cohort, HDL: high-density lipoprotein, HISB: Health Interview Survey of Barcelona, IADL: instrumental activity of daily living limitations, KMCC: Korean Multi-center Cancer Cohort study, KYCHS: Kiryat Yovel Community Health Study, MCS: Mental Component Summary, MEC: Mini-Examen Cognoscitivo score, MVPA, moderate-to-vigorous physical activity, PCS: Physical Component Summary, SF-36: Medical Outcomes Study 36-item Short Form Survey, SHLSE: Survey of Health and Living Status of the Elderly, SOF: Study of Osteoporotic Fractures, SWMHS: Shanghai Women’s and Men’s Health Studies

Table S3 Summary of the included studies and results from meta-regression

	Nighttime sleep				24-hour sleep[b]			
	**N[a]**	**Participants**	**References**	***P*[c]**	**N[a]**	**Participants**	**References**	***P*[c]**
Overall	36	1,526,609	1,3-4,6-9,11,13-18,20-24,27,30,32-35		19	903,727	2,5,8,10,12,16,19,25-26,28-29,31,33	
Subjects without cardiovascular diseases and cancer at baseline	10	329,420	9,15,27,30,32-33		4	341,530	26,28,31	
Country				0.16				0.05
Asia	18	158,270	1,4,8-9,13,15,17-18,21-22,27		6	177,400	8,25,29,31	
Europe	9	100,024	3,7,11,23,30,32,35		7	31,861	2,5,12,19	
USA	9	1,268,315	6,14,16,20,24,33-34		5	466,651	10,16,26,33	
Sex				0.27				0.35
Men	12	556,127	4,6-9,13,15,17,23-24,27,32		6	124,591	5,8,12,19,26,31	
Women	12	732,420	4,6-9,13,15-17,24,27,32		8	247,786	5,8,10,12,16,19,26,31	
Both	12	238,062	1,3,11,14,18,20-22,30,33-35		5	531,350	2,25,28-29,33	
Mean age				0.74				0.32
>60 years	19	299,741	1,3,8,13,15-17,20-22,24,30,33-35		11	667,634	The others	
≤60 years	17	1,226,868	The others		8	236,093	5,10,12,29,31	
Follow-up duration				0.22				0.05
≥10 years	20	347,375	3-4,7-8,11,14-15,18,21,23-24,30,32		11	536,470	The others	

<10 years	16	1,179,234	The others		8	367,257	[2,16,19,29,31]	
Sleep assessment				0.55				0.08
Self-administered	21	1,468,468	[1,4,6-8,11,15-17,24,30,32-33]		15	776,206	The others	
Interviewer-administered	15	58,141	The others		4	127,521	[2,29,31]	
Adjusted for ...								
Education/socioeconomic status				0.40				0.97
Yes	27	1,492,775	The others		17	812,657	The others	
No	9	33,834	[1,4,7,16,27,35]		2	91,070	[10,16]	
Smoking				0.90				0.61
Yes	28	1,506,044	The others		18	902,508	The others	
No	8	20,565	[1,3-4,7,21,32]		1	1,219	[2]	
Alcohol				0.89				0.61
Yes	24	377,585	The others		18	902,508	The others	
No	12	1,149,024	[1,3-4,6-7,14,21,32,35]		1	1,219	[2]	
Physical activity				0.95				0.83
Yes	25	1,489,383	The others		17	889,344	The others	
No	11	37,226	[1,3-4,7,9,22,32]		2	14,383	[2,29]	
Health status[d]				0.48				0.21
Yes	10	1,273,266	[6,8,11,14,20-21,23,33]		10	530,876	The others	
No	26	253,343	The others		9	372,851	[10,12,16,26,29,31]	
Blood pressure				0.77				0.52
Yes	22	1,291,906	The others		12	301,865	The others	

No	14	234,703	[1,7,13,21,27,30,32-35]		7	601,862	[2,12,28,31,33]	
Body mass index				0.83				0.61
Yes	26	1,502,834	The others		18	902,508	The others	
No	10	23,775	[1,4,7,21,24,32]		1	1,219	[2]	
Preexisting chronic diseases				0.22				0.42
Yes	32	1,518,909	The others		14	875,686	The others	
No	4	7,700	[1,7,20]		5	28,041	[2,5,12]	
Sleep disorders/ siesta				0.74				0.20
Yes	13	1,158,,557	[4,6,14,16,20,22,24,32,35]		6	96,732	[8,10,16,19]	
No	23	368,052	The others		13	806,995	The others	
Depression/ mental health				0.44				0.23
Yes	15	145,756	[13-17,20-22,24,34-35]		5	144,146	[10,16,19,25]	
No	21	1,380,853	The others		14	759,581	The others	
Study quality				0.40				0.51
7-8 stars	13	339,040	[3,11,15,18,21,23,27,30,32-33]		10	653,902	[10,12,25-26,29,31,33]	
4-6 stars	23	1,187,569	The others		9	249,825	The others	

a: N: number of studies (gender-specific studies).

b: there is a study conducted in Australia.

c: P values from meta-regression with a permute test of 1000.

d: health status (self-reported health status or physical functioning)

The search syntax is as follows: *((((((prospective) OR cohort) OR longitudinal) OR follow-up)) AND ((death) OR mortality)) AND sleep)*, without restrictions. The titles/abstracts of the articles were screened for the following inclusion criteria: sleep duration and all-cause mortality as the potential exposure and outcome of interest, conducted in humans and adults, prospective cohort studies and original articles.

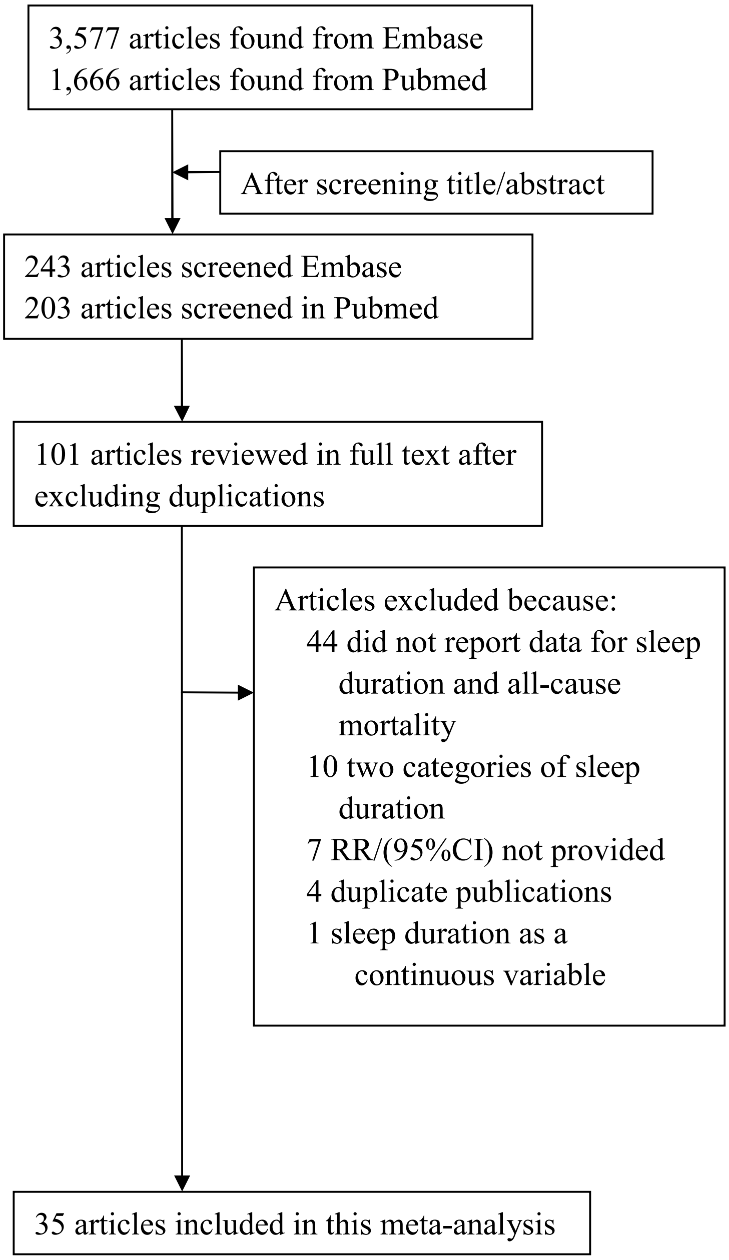

Figure S1 The flow chart for detailed steps of literature search

The following papers were excluded because of: two categories of sleep duration[36-45], RR/(95%CI) not provided[46-52], duplicate publications[53-54], and sleep duration as a continuous variable[55].

References

1. Tsubono, Y., Fukao, A. & Hisamichi, S. Health practices and mortality in a rural Japanese population. *Tohoku J Exp Med* **171**, 339-348 (1993).
2. Ruigomez, A., Alonso, J. & Anto, J. M. Relationship of health behaviours to five-year mortality in an elderly cohort. *Age Ageing* **24**, 113-119 (1995).
3. Gale, C. & Martyn, C. Larks and owls and health, wealth, and wisdom. *BMJ* **317**, 1675-1677 (1998).
4. Kojima, M. *et al.* Sleep patterns and total mortality: a 12-year follow-up study in Japan. *J Epidemiol* **10**, 87-93 (2000).
5. Heslop, P., Smith, G. D., Metcalfe, C., Macleod, J. & Hart, C. Sleep duration and mortality: The effect of short or long sleep duration on cardiovascular and all-cause mortality in working men and women. *Sleep Med* **3**, 305-314 (2002).
6. Kripke, D. F., Garfinkel, L., Wingard, D. L., Klauber, M. R. & Marler, M. R. Mortality associated with sleep duration and insomnia. *Arch Gen Psychiatry* **59**, 131-136 (2002).
7. Mallon, L., Broman, J. E. & Hetta, J. Sleep complaints predict coronary artery disease mortality in males: a 12-year follow-up study of a middle-aged Swedish population. *J Intern Med* **251**, 207-216 (2002).
8. Burazeri, G., Gofin, J. & Kark, J. D. Over 8 hours of sleep--marker of increased mortality in Mediterranean population: follow-up population study. *Croat Med J* **44**, 193-198 (2003).
9. Amagai, Y. *et al.* Sleep duration and mortality in Japan: the Jichi Medical School Cohort Study. *J Epidemiol* **14**, 124-128 (2004).
10. Patel, S. R. *et al.* A prospective study of sleep duration and mortality risk in women. *Sleep* **27**, 440-444 (2004).
11. Ferrie, J. E. *et al.* A prospective study of change in sleep duration: associations with mortality in the Whitehall II cohort. *Sleep* **30**, 1659-1666 (2007).
12. Hublin, C., Partinen, M., Koskenvuo, M. & Kaprio, J. Sleep and mortality: a population-based 22-year follow-up study. *Sleep* **30**, 1245-1253 (2007).
13. Lan, T. Y., Lan, T. H., Wen, C. P., Lin, Y. H. & Chuang, Y. L. Nighttime sleep, Chinese afternoon nap, and mortality in the elderly. *Sleep* **30**, 1105-1110 (2007).
14. Gangwisch, J. E. *et al.* Sleep duration associated with mortality in elderly, but not middle-aged, adults in a large US sample. *Sleep* **31**, 1087-1096 (2008).
15. Ikehara, S. *et al.* Association of sleep duration with mortality from cardiovascular disease and other causes for Japanese men and women: the JACC study. *Sleep* **32**, 295-301 (2009).
16. Stone, K. L. *et al.* Self-reported sleep and nap habits and risk of mortality in a large cohort of older women. *J Am Geriatr Soc* **57**, 604-611 (2009).
17. Suzuki, E. *et al.* Sleep duration, sleep quality and cardiovascular disease mortality among the elderly: a population-based cohort study. *Prev Med* **49**, 135-141 (2009).
18. Chien, K. L. *et al.* Habitual sleep duration and insomnia and the risk of cardiovascular events and all-cause death: report from a community-based cohort. *Sleep* **33**, 177-184 (2010).
19. Mesas, A. E., Lopez-Garcia, E., Leon-Munoz, L. M., Guallar-Castillon, P. & Rodriguez-Artalejo, F. Sleep duration and mortality according to health status in older

adults. *J Am Geriatr Soc* **58**, 1870-1877 (2010).

20. Castro-Costa, E. *et al.* Association between sleep duration and all-cause mortality in old age: 9-year follow-up of the Bambui Cohort Study, Brazil. *J Sleep Res* **20**, 303-310 (2011).
21. Cohen-Mansfield, J. & Perach, R. Sleep duration, nap habits, and mortality in older persons. *Sleep* **35**, 1003-1009 (2012).
22. Chen, H. C., Su, T. P. & Chou, P. A nine-year follow-up study of sleep patterns and mortality in community-dwelling older adults in Taiwan. *Sleep* **36**, 1187-1198 (2013).
23. Garde, A. H., Hansen, A. M., Holtermann, A., Gyntelberg, F. & Suadicani, P. Sleep duration and ischemic heart disease and all-cause mortality: prospective cohort study on effects of tranquilizers/hypnotics and perceived stress. *Scand J Work Environ Health* **39**, 550-558 (2013).
24. Jung, K. I., Song, C. H., Ancoli-Israel, S. & Barrett-Connor, E. Gender differences in nighttime sleep and daytime napping as predictors of mortality in older adults: the Rancho Bernardo study. *Sleep Med* **14**, 12-19 (2013).
25. Kakizaki, M. *et al.* Long sleep duration and cause-specific mortality according to physical function and self-rated health: the Ohsaki Cohort Study. *J Sleep Res* **22**, 209-216 (2013).
26. Kim, Y. *et al.* Insufficient and excessive amounts of sleep increase the risk of premature death from cardiovascular and other diseases: the Multiethnic Cohort Study. *Prev Med* **57**, 377-385 (2013).
27. Li, Y., Sato, Y. & Yamaguchi, N. Potential biochemical pathways for the relationship between sleep duration and mortality. *Sleep Med* **14**, 98-104 (2013).
28. Magee, C. A., Holliday, E. G., Attia, J., Kritharides, L. & Banks, E. Investigation of the relationship between sleep duration, all-cause mortality, and preexisting disease. *Sleep Med* **14**, 591-596 (2013).
29. Yeo, Y. *et al.* A prospective cohort study on the relationship of sleep duration with all-cause and disease-specific mortality in the Korean Multi-center Cancer Cohort study. *J Prev Med Public Health* **46**, 271-281 (2013).
30. Bellavia, A., Akerstedt, T., Bottai, M., Wolk, A. & Orsini, N. Sleep duration and survival percentiles across categories of physical activity. *Am J Epidemiol* **179**, 484-491 (2014).
31. Cai, H. *et al.* Sleep Duration and Mortality: A Prospective Study of 113 138 Middle-Aged and Elderly Chinese Men and Women. *Sleep* (2014).
32. Rod, N. H. *et al.* The joint effect of sleep duration and disturbed sleep on cause-specific mortality: results from the Whitehall II cohort study. *PLoS One* **9**, e91965 (2014).
33. Xiao, Q., Keadle, S. K., Hollenbeck, A. R. & Matthews, C. E. Sleep duration and total and cause-specific mortality in a large US cohort: interrelationships with physical activity, sedentary behavior, and body mass index. *Am J Epidemiol* **180**, 997-1006 (2014).
34. Hall, M. H. *et al.* Association between Sleep Duration and Mortality Is Mediated by Markers of Inflammation and Health in Older Adults: The Health, Aging and Body Composition Study. *Sleep* **38**, 189-195 (2015).

35. Zuurbier, L. A. *et al.* Fragmentation and stability of circadian activity rhythms predict mortality: the Rotterdam study. *Am J Epidemiol* **181**, 54-63 (2015).
36. Dew, M. A. *et al.* Healthy older adults' sleep predicts all-cause mortality at 4 to 19 years of follow-up. *Psychosom Med* **65**, 63-73 (2003).
37. Vgontzas, A. N. *et al.* Insomnia with short sleep duration and mortality: the Penn State cohort. *Sleep* **33**, 1159-1164 (2010).
38. Eguchi, E. *et al.* Healthy lifestyle behaviours and cardiovascular mortality among Japanese men and women: the Japan collaborative cohort study. *Eur Heart J* **33**, 467-477 (2012).
39. Ensrud, K. E. *et al.* Sleep disturbances and risk of frailty and mortality in older men. *Sleep Med* **13**, 1217-1225 (2012).
40. Martinez-Gomez, D., Guallar-Castillon, P., Leon-Munoz, L. M., Lopez-Garcia, E. & Rodriguez-Artalejo, F. Combined impact of traditional and non-traditional health behaviors on mortality: a national prospective cohort study in Spanish older adults. *BMC Med* **11**, 47 (2013).
41. Duggan, K. A., Reynolds, C. A., Kern, M. L. & Friedman, H. S. Childhood sleep duration and lifelong mortality risk. *Health Psychol* **33**, 1195-1203 (2014).
42. Lee, J. S. *et al.* Long sleep duration is associated with higher mortality in older people independent of frailty: a 5-year cohort study. *J Am Med Dir Assoc* **15**, 649-654 (2014).
43. Sivertsen, B. *et al.* Midlife insomnia and subsequent mortality: the Hordaland health study. *BMC Public Health* **14**, 720 (2014).
44. Branch, L. G. & Jette, A. M. Personal health practices and mortality among the elderly. *Am J Public Health* **74**, 1126-1129 (1984).
45. Kaplan, G. A., Seeman, T. E., Cohen, R. D., Knudsen, L. P. & Guralnik, J. Mortality among the elderly in the Alameda County Study: behavioral and demographic risk factors. *Am J Public Health* **77**, 307-312 (1987).
46. Kronholm, E., Laatikainen, T., Peltonen, M., Sippola, R. & Partonen, T. Self-reported sleep duration, all-cause mortality, cardiovascular mortality and morbidity in Finland. *Sleep Med* **12**, 215-221 (2011).
47. Hammond, E. C. Some Preliminary Findings on Physical Complaints from a Prospective Study of 1,064,004 Men and Women. *Am J Public Health Nations Health* **54**, 11-23 (1964).
48. Belloc, N. B. Relationship of health practices and mortality. *Prev Med* **2**, 67-81 (1973).
49. Kripke, D. F., Simons, R. N., Garfinkel, L. & Hammond, E. C. Short and long sleep and sleeping pills. Is increased mortality associated? *Arch Gen Psychiatry* **36**, 103-116 (1979).
50. Breslow, L. & Enstrom, J. E. Persistence of health habits and their relationship to mortality. *Prev Med* **9**, 469-483 (1980).
51. Wingard, D. L. & Berkman, L. F. Mortality risk associated with sleeping patterns among adults. *Sleep* **6**, 102-107 (1983).
52. Kripke, D. F., Langer, R. D., Elliott, J. A., Klauber, M. R. & Rex, K. M. Mortality related to actigraphic long and short sleep. *Sleep Med* **12**, 28-33 (2011).
53. Tamakoshi, A. & Ohno, Y. Self-reported sleep duration as a predictor of all-cause

mortality: results from the JACC study, Japan. *Sleep* **27**, 51-54 (2004).
54. Suzuki, K. Health conditions and mortality in the Japan Collaborative Cohort Study for Evaluation of Cancer (JACC). *Asian Pac J Cancer Prev* **8 Suppl**, 25-34 (2007).
55. Pollak, C. P., Perlick, D., Linsner, J. P., Wenston, J. & Hsieh, F. Sleep problems in the community elderly as predictors of death and nursing home placement. *J Community Health* **15**, 123-135 (1990).
